# Minimal invasive management of bladder neck contracture using Allium round posterior stent: the long-term results

**DOI:** 10.1016/j.prnil.2021.05.004

**Published:** 2021-05-29

**Authors:** Kerem Teke, Efe Bosnali, Onder Kara, Murat Ustuner, Ibrahim E. Avci, Mustafa M. Culha

**Affiliations:** a Department of Urology, Kocaeli University School of Medicine, Kocaeli, Turkey; b Department of Urology, Derince Training and Research Hospital, Kocaeli, Turkey

**Keywords:** Allium round posterior stent, Bladder neck contracture, Clinical efficacy, Long-term follow-up, Urethral stent

## Abstract

**Background:**

The purpose of this study was to assess the long-term clinical efficacy of temporary, Allium round posterior stent (RPS) used for treatment of recurrent bladder neck contracture (BNC).

**Methods:**

Records of 42 patients with recurrent BNC who underwent Allium RPS placement after bladder neck incision, between 2009 and 2019, were analyzed. After stent removal, the success criteria for Allium RPS treatment were defined as: no evidence of stricture on urethrogram or endoscopy; more than 12 ml/sec of urinary peak flow; and no recurrent urinary tract infections. Based on clinical success, patients were divided into two groups and compared. Clinical success was evaluated with particular regard to stent indwelling time and contracture etiology.

**Results:**

The mean ± standard deviation age, stricture length, and indwelling time were 66.7 ± 9 years, 2.4 ± 1.4 cm, and 7.7 ± 2.2 months, respectively. Median (range) follow-up was 59 (8–73) months. The etiologies of BNC in this cohort were 57.1% retropubic radical prostatectomy; and 42.9% transurethral resection of prostate. Overall clinical success was achieved in 64.3% and the success rates did not differ by etiology. The success rates were 54.2% and 77.8% (*P* = 0.118) for retropubic radical prostatectomy and transurethral resection of prostate, respectively. Longer indwelling time (8–14 vs 3–7, months) was significantly associated with clinical success (78.3% vs 47.4%, *P* = 0.040).

**Conclusion:**

Our data suggest that better clinical success was associated with longer indwelling time for stent in BNC treatment. In BNC management, Allium RPS treatment may be considered since its clinical efficacy is acceptable and tolerable.

## 1 Introduction

Bladder neck contracture (BNC) is a rare but challenging entity and usually occurs as a complication after the surgical treatment of benign and malignant prostatic diseases. BNC may occur after transurethral resection of the prostate (TUR-P) with a reported frequency of 0–4.9% and the reported prevalence varied between 8.8 and 33% after radical prostatectomy.^1^, ^2^, ^3^, ^4^, ^5^ Although BNC can occur more commonly after radical prostatectomy compared to TUR-P, the prevalence has decreased to a rate of 1.1% with surgical advancements, including robot-assisted laparoscopic prostatectomy.^6^^,^^7^ The incidence likely depends on the surgical technique used. Potential risk factors for BNC following TUR-P have been proposed to be a low adenoma weight, extensive resection of the bladder neck, and use of a large resecting loop.^8^^,^^9^ Urinary extravasation, asymptomatic bacteriuria, previous prostatectomy, membranous urethra, or bladder neck ischemia may be further risk factors for BNC after radical prostatectomy.^6^^,^^10^ BNC typically presents clinically within 12 months of radical prostatectomy,^11^ while the development period for BNC after TUR-P is nearly 18 months.^8^ Significant morbidities are related to BNC, including retention, incontinence, infection of the urinary system, and the need for a secondary invasive procedure.^4^^,^^12^ However, even though the prevalence of BNC has been reduced dramatically, when it does occur, BNC reduces the patients’ quality of life, and endoscopic procedures such as transurethral bladder neck incision and metal sound dilation might be required.^7^^,^^13^ The development of bladder outlet obstruction due to BNC may lead to detrusor and renal failure, recurrent urinary tract infections, urinary retention, hematuria, and bladder stones.^9^ Albeit the complex and recalcitrant nature of BNC presents a clinical challenge to the urologist, it can be managed with dilation, bladder neck incision, or transurethral resection, and open reconstruction.^14^ However, there are no clear guidelines currently available for management. Furthermore, in recent years, urethral stent interventions have been used to treat a range of urethral strictures.^9^^,^^15^

In the present study, patients with recurrent BNC who were treated using Allium round posterior stent (RPS) were retrospectively evaluated. The 10-year experience of a single surgeon using Allium RPS for BNC treatment was presented. This cohort could be the largest series of use of Allium RPS for recurrent BNC. Importantly, the data presented includes long-term clinical outcomes.

## 2 Materials and methods

### 2.1 Patients

The patients with recurrent BNC were endoscopically treated with Allium (Allium LTD, Caesarea, Israel) RPS by a single surgeon (MMC) between 2009 and 2019 in a tertiary center hospital in Kocaeli, Turkey. Adult patients with recurrent BNC who had complication after prostate surgery were included. Exclusion criteria for the study were patients who had strictures in other regions of the urethra; a history of pelvic malignancy other than prostate cancer; and history of pelvic radiation therapy. Preoperative demographics and clinical characteristics were collected including age, maximum urinary flow rate, number of previous bladder neck incisions or dilatations, and time of recurrence after the last stricture treatment. Patients were also evaluated with retrograde urethrogram and uroflowmetry. Post-micturition residual urine was analyzed with suprapubic ultrasonography; all patients with BNC had greater than 150 ml of volume in the bladder. All patients provided informed consent before Allium RPS placement. Bladder neck incision was performed before stent placement. Stricture length was estimated during urethroscopy. Stricture etiology and prior treatments were documented. The study protocol was approved by the Local Ethics Committee (Approval number: GOKAEK-2021/1.01).

### 2.2 Stents and operation procedure

**Fig. 1:**
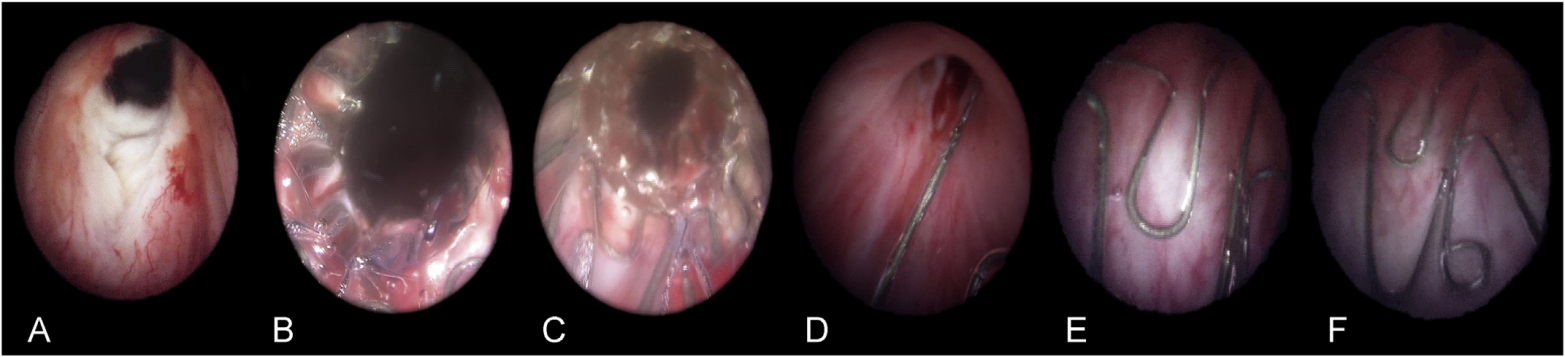
Cystoscopic images of a patient with bladder neck contracture treated with Allium round posterior stent (RPS): **A)** incised bladder neck; **B)** proximal and **C)** distal segment of Allium RPS inserted into bladder neck; **D)** Trans-sphincteric wire provides connection between the Allium RPS and its anchor; **E)** the stent anchor positioned under the external urethral sphincter level; **F)** the “O" shaped hook, integral to the anchor, which is used for extracting the Allium RPS.

The Allium RPS, designed for the treatment of BNC, is fully covered by a co-polymer, which prevents tissue in-growth and reduces encrustations and calcifications.^16^ Because of its round shape and placement in the posterior urethra, this stent type has named as RPS. It has a connecting trans-sphincteric wire, and this part is designed to minimize sphincteric dysfunction. The Allium RPS has an extremely strong and flexible body, provides resistance to occluded passages with minimal irritation and high radial force, and has a large caliber (45 Fr). Its length is 30 mm or 40 mm. The Allium RPS is intended for temporary use only so that, during the indwelling period, the stent acts as a mold and allows the growth of a functional passage. Stent insertion is performed using a gun-like delivery system on which the stent is mounted and deployment is gradual rather than sudden. The first step is bladder neck incision. Bladder neck incision at the 5 and 7 o'clock position was performed with monopolar or bipolar electrode Collins knife using 24 Fr/26 Fr resectoscope before stent placement. Subsequently, after measuring the length of the stricture, the stent was placed endoscopically under visual guidance within the bladder neck and the stent anchor was adjusted so that it was positioned just distal of the external urethral sphincter (Fig. 1A–E). All the procedures were carried out under spinal or general anesthesia. Once inserted into the bladder neck with the aid of the special inserter, the stent is released to allow its self-expansion. A feature of this Allium RPS is that it is capable of being unraveled into a thread-like strip, enabling a nontraumatic removal (Fig. 1F). The indwelling time for the stents was planned to be 12 months as described our another study used urethral stent.^17^ Progressive decrease of urinary peak flow rate during this period, recurrent urinary infection, and/or stent migration were removal criteria. Clinically stent migration was considered when the patient suddenly encountered difficulty to urinate and subsequently stent was removed by performing cystoscopy.

### 2.3 Assessment of quality of life during stent indwelling period and follow-up after stent removal

For the evaluation of the quality of life, any report of dysuria, incontinence, or discomfort while sitting was also recorded for each patient during the indwelling period. Dysuria is defined by degree as mild (pain that does not need any analgesics); moderate (pain that needs analgesics); and severe (pain that persists despite analgesics). Clinical success criteria were no evidence of stricture on urethrogram or endoscopy six months after stent removal, urinary peak flow greater than 12 ml/sec, and no recurrent urinary tract infection in the follow-up. In addition, for clinical success, there should be no requirement for further procedures, such as dilatation or catheterization, after stent removal. For the follow-up protocol, urinary peak flow rates were estimated at 3, 6, 9, and 12 months after stent removal and urine cultures were taken at each of these visits. At the end of one year of clinical follow-up, telephone follow-up was performed every six months. The correlation between the success rate of stent treatment and the indwelling period, divided into shorter (up to median indwelling period) and longer time (more than median indwelling period), was assessed. Additionally, the effect of BNC etiology on the Allium RPS treatment success was also investigated. Statistical analysis was performed using SPSS, version 13.0 (IBM Inc., Armonk, NY, USA). Normally distributed data are presented as mean ± standard deviation and nonparametric data are presented as median and range. The paired t-test and Mann-Whitney U test were used to analyze and compare the groups.

## 3 Results

The cohort consisted of 42 patients in whom Allium 48 RPS were placed. The mean operation time for all 48 RPS was 43 ± 12 min. The mean age of patients was 66.7 ± 9 years. The cause of BNC was TUR-P in 18 (mono-polar n = 8, bi-polar n = 10) and open retropubic radical prostatectomy (RRP) in 24 patients. All of the patients who underwent RRP had PSA concentration <0.04 ng/mL before stent placement. The mean length of the stricture was 2.4 ± 1.4 cm. The number of patients undergoing one or more bladder neck incisions or dilatations prior to stenting were one n = 9, two n = 19, and more than two n = 14. The estimated time of development of BNC recurrence from the last treatment was one month for seven patients, one to two months for 12 patients, two to three months for 11 patients, and more than three months for the other 12 patients. The mean time of BNC recurrence after the last treatment of BNC was 47 ± 21 days. Demographic and clinical data of the patients are summarized in Table 1.

**Table 1:** The demographics and clinical characteristics of patients with bladder neck contracture

Variables		
Patients, (n)	42	
Stents, (n)	48	
Age, mean ± SD (years)	66.7 ± 9	
Length of stricture, mean ± SD (cm)	2.4 (2.4 – 1.4)	
Indwelling period of stent, median (range), (months)	7 (3 – 14)	
Follow-up after stent removal, median (range), (months)	59 (8 – 73)	
Allium round posterior stent (RPS) size (n, %)		
3 cm	44 (91.7%)	
4 cm	4 (8.3%)	
Etiology (n, %)		
Transurethral resection of the prostate	18 (42.9%)	
Monopolar	8 (19.0%)	
Bipolar	10 (23.8%)	
Open retropubic radical prostatectomy	24 (57.1%)	
Number of bladder neck dilatations or incisions before the procedure, per patient (n, %)		
1	9 (21.4%)	
2	19 (45.2%)	
≥3	14 (33.3%)	
The patients' recurrence time of stricture after last bladder neck dilatations or incisions (months)		
<1	7 (16.7%)	
1-2	12 (28.6%)	
2-3	11 (26.2%)	
≥3	12 (28.6%)	
Preop urinary peak flow rate, mean ± SD, ml/sec	3.1 ± 1.2 ml/sec^a^	*P* < 0.001
Postop urinary peak flow rate, mean ± SD, ml/sec^b^	14.2 ± 4.7 ml/sec^a^	

SD, standard deviation.

a Paired t-test was used for comparison of dependent samples. A *P* level <0.05 was considered statistically significant.

b The measurement of all patients' urinary peak flow rate at one week after primary Allium RPS placement. The urinary peak flow data regarding new stents replaced were not included for this comparison.

Allium RPS stents were positioned correctly into the bladder neck in all patients. No adverse events related to the stent placement or the procedure were recorded. All patients were discharged after voiding satisfactorily. Whereas the mean urinary peak flow rate was 3.1 ± 1.2 ml/sec before the procedure, the mean urinary peak flow rate one week after the primary stent placement was 14.2 ± 4.7 ml/sec (*P* < 0.001, Table 1).

Eighteen (42.9%) patients complained of mild early urinary stress incontinence (defined as the need for ≤1 pad/day) postoperatively up to one month. Mild late urinary stress incontinence (after one month) continued during the indwelling period of Allium RPS in 15 (35.7%) patients. All stents were easily removed 3 to 14 months after implantation (median 7 months) under local anesthesia. There was no evident postprocedure complication. Five (11.9%) Allium RPS migrated into the bladder and one (2.4%) Allium RPS migrated distally into the membranous urethra up to one month postoperatively. All migrated stents were replaced with new stents immediately after removal of the malpositioned stent. Stent migration was again observed at third and fourth months postoperatively, in four patients, in whom this complication had previously occurred. In these patients, the stents were removed. The reasons for early stent removal in the period from 4 to the end of 7 months after implantation were migration in 14 patients and progressive decreasing urinary peak flow in one. Of the remaining 23 patients who had a longer indwelling time (8 to 14 months, median 9 months), in four patients the stent migrated by the eighth month (n = 2) and twelfth month (n = 2), and acute symptomatic urinary infection was observed in two. When the macroscopic structure of stents removed was examined, there was no stone formation on the stents, while encrustation formation was observed in two stents obtained from patients with acute symptomatic urinary infection.

Quality of life assessment showed that the rate of mild dysuria and discomfort on sitting that occurred up to a month after stent placement was 9.5% (n = 4) and 4.8% (n = 2), respectively. Of the adverse events up to one month, mild dysuria was completely resolved in two patients (4.8%). Of six patients who were replaced with the stents again, five did not have any complaints regarding the quality-of-life parameters until new stent replacement. However, one of six had discomfort in sitting, which is fully recovered after stent change. After one month, up to stents removal, persistent mild dysuria in two patients (4.8%) and discomfort on sitting in one patient were reported. Moderate or severe dysuria was not observed during the indwelling period, while moderate dysuria in 7 patients (16.7%) was encountered at only migration time. All complications and results of the quality of life assessment during the indwelling period are presented in Table 2.

**Table 2:** The complications and quality of life assessments of 48 Allium round posterior stent (RPS) during the indwelling period in 42 patients with bladder neck contracture

Period	Complication (n, %)	Quality of life parameters
Up to one month	Migration (6, 14.3%)^a^ Mild early incontinence (18, 42.9%)	Mild dysuria (4, 9.5%) Discomfort in sitting (2, 4.8%)
After one month (1 to 14 months)	Mild late incontinence (15, 35.7%) Migration (22, 52.4%) Progressive decreased urinary flow (1, 2.4%) Acute symptomatic urinary infection (2, 4.8%)	Mild dysuria (2, 4.8%) Discomfort in sitting (1, 2.4%)

a All six migrated Allium RPS were replaced with new ones.

Median (range) follow-up was 59 (8–73) months after stent removal. Mean urinary peak flow rates at 3, 6, 9, and 12 months after stent removal were 14.7 ± 6.7 ml/sec, 12.2 ± 4.3 ml/sec, 13.4 ± 5.3 ml/sec, and 12.7 ± 5.7 ml/sec, respectively. There was no biochemical recurrence in patients who had undergone RRP during the follow-up. As shown in Table 3, overall clinical success was observed in 27 (64.3%) patients. Longer indwelling time (>7 months) was significantly associated with higher clinical success (78.3% vs 47.4%, *P* = 0.040), compared with a shorter indwelling period (≤7 months, median 4 months). When assessing the association of clinical success with etiology (TUR-P vs RRP), clinical success after stent removal tended to be higher in patients with a TUR-P etiology but this was not significant (77.8% vs 54.2%, *P* = 0.118). The urethral dilatation was recommended to the patients with RPS treatment failure in this study cohort.

**Table 3:** The comparison of clinical success by Allium round posterior stent (RPS) indwelling time and etiology of bladder neck contracture

n = 42	Success (n = 27, 64.3%)	Nonsuccess (n = 15, 35.7%)	*P*
Allium RPS indwelling time (months), n			0.040^a^
Shorter period (≤7 months), n = 19	9 (47.4%)	10 (52.6%)	
Longer period (>7 months), n = 23	18 (78.3%)	5 (21.7%)	
Etiology, n			0.118
Transurethral resection of the prostate, n = 18	14 (77.8%)	4 (22.2%)	
Open retropubic radical prostatectomy, n = 24	13 (54.2%)	11 (45.8%)	

a Mann-Whitney U test was used for comparison of independent samples. A *P* level <0.05 was considered statistically significant.

## 4 Discussion

The first stent reported in the literature that was used for the management of BNC was the UroLume “endoprosthesis” in 1989 (American Medical Systems, Minnesota, USA).^15^^,^^18^ Since then, urethral stents have been used as an alternative minimally invasive treatment option in patients with urethral strictures who are not suitable for urethroplasty, in patients with catheter due to benign prostatic obstruction, in patients with an obstruction due to prostate cancer, and also in patients who are candidates for surgical sphincterotomy due to spinal trauma.^15^ Unfortunately, there were short- and long-term complications associated with permanent stents such as UroLume,^19^^,^^20^ which has resulted in persisting prejudice against the use of permanent stents. However, temporary stents have also been used for urethral stricture. For BNC, no definitive treatment choice has been shown to be optimal and therefore none is recommended. However, open or robotic reconstruction of the bladder neck and minimally invasive approaches including urethral dilatation, urethral incision, and injection of antiproliferative agents (Mitomycin C or steroids) may be performed. Given the lack of consensus for treatment of BNC, the associated literature is limited and reported clinical success is remarkably variable. In this study, the single-center, single operator long-term experience of Allium RPS for treatment of BNC is presented. The results, including clinical response, complications experienced, and patient satisfaction, are reviewed and discussed in light of other published treatment choices.

The frequency, etiology, and complexity of BNC after surgery vary depending on what treatment was used.^2^^,^^21^ For instance, patients who underwent radical prostatectomy for prostate cancer are likely to develop BNC due to technical factors at the level of the vesicourethral anastomosis,^3^ while extreme cauterization with loop during TUR-P at the bladder neck might result in BNC after treatment for benign prostatic hyperplasia. In our series, all patients had BNC that occurred after RRP and TUR-P. Moreover, BNC is associated with significant morbidity and represents a major clinical challenge, which may be overcome by various treatment modalities. First line treatments usually include minimally invasive methods as an initial step in BNC management. The American Urologic Association guidelines recommended that surgeons perform a dilation, bladder neck incision, or transurethral resection for BNC after an endoscopic prostate procedure.^22^ Although transurethral incision of the bladder neck has been recommended as a successful first-line treatment for BNC, in some patients BNC recurs rapidly and is resistant to further therapy. Transurethral bladder neck incision may be performed using cold-knife, electrocautery, laser, hot-knife, and loop resection.^21^^,^^23^ Another treatment approach, dilatation of the urethral stricture, may be carried out by the physician or self-administered by the patient. However, periodic self-catheterization performed after bladder neck dilatation has been reported to be associated with a reduction in patients' quality of life, especially due to pain and difficulty.^24^ Moreover, incision and dilatation could be performed together.^21^ A few studies have reported promising success rates with the use of antiproliferative agents, such as mitomycin-C (92.9%) or steroids (83%), injected into BNC tissues after incision or resection.^25^^,^^26^

In addition to minimally invasive treatment choices, bladder neck reconstruction is technically complex and also has greater complication rates including stress urinary incontinence. Most published series of open bladder neck reconstructions are limited by short follow-up and small study groups. For reconstruction, although there are abdominoperineal, perineal, and transpubic approaches,^27^^,^^28^ recently the Y–V Plasty procedure, which can be performed with robot-assisted laparoscopy, was demonstrated and promising results have been published.^29^^,^^30^ Clinical outcomes in all these treatment modalities report very variable success or recurrence rates. In the present cohort all patients had been treated before, more than once, with minimally invasive treatment modalities. Our success rate was 78.3% in the patients treated with Allium RPS after a long-term indwelling period (median 9 months). It is also reassuring that none of these patients had any evidence of, not reported, any recurrence during follow-up.

In 2005 Anger et al reported that severe anastomotic contractures after radical prostatectomy may be managed using a minimally invasive approach using UroLume stenting with acceptable outcomes.^31^ However, Breyer and McAninch claimed that open surgical reconstruction for BNC after radical prostatectomy was superior to UroLume stenting in patients with reasonable life expectancy for prostate cancer due to the successful rehabilitation in 7 of 10 patients with complex vesicourethral stenosis.^13^ Moreover, UroLume stents are not temporary. Hence, use of this permanent stent may lead to long-term complications, as detailed in a case which reported complete overgrowth of the stent and the presence of encrusted tissue which occluded the distal part of the stent.^32^ Unlike permanent stents, such as the UroLume, treatment response evaluation after removal of the Allium temporary stent in our study showed that the clinical success was 64.3% for the whole cohort on long-term follow-up (median 59 months). In particular, our data highlighted the benefit of longer indwelling time. With respect to this, it might be associated with between prevention of BNC formation and longer stent indwelling. We consider that the stent placement after bladder neck incision might prevent the recurrence of BNC by providing both active (bladder neck incision) and passive (by blocking luminal obliteration against contracture formation depending stent indwelling time) treatment. For this reason, we propose the need for development of a new generation of temporary stents that would have both a longer indwelling time and result in fewer complications, such as urinary incontinence and stent migration. Our encouraging results regarding Allium RPS might be kept in the mind as a minimally invasive treatment approach for the management of BNC.

Recently, Wen et al reported that a patient with a BNC after TUR-P was treated with an expandable metal stent that remained *in situ* for 24 months. These authors again reported that the patient did not have any complaints over this period.^9^ Additionally, our report showed that the discomforts of our patients in the first month were easily tolerable during quality of life assessment.

The limitations of this study were as follows. First, the etiology of BNC was restricted to post RRP or TUR-P. Patients undergoing other prostatectomy procedures, including robotic-assisted laparoscopic or laparoscopic radical prostatectomy and holmium laser prostatic enucleation of prostate were excluded. Second, there was no assessment of sexual function. We did not evaluate sexual and ejaculatory function in this study because most of these patients already had secondary sexual and ejaculatory dysfunctions due to previous prostatic operations. Third, after the first year post-op follow-up was limited to telephone conversations. Fourth, there was no BNC patient group, which was not treated with a stent in this study, to compare patients treated with the Allium RPS. Finally, the study was retrospective in design.

## 5 Conclusion

In conclusion, to the best of our knowledge, this study presents the largest series of patients treated with Allium RPS for BNC. We believe that this approach, which is a minimally invasive method, is a treatment modality that should be more widely considered since it does not significantly impair the quality of life and its clinical efficacy is acceptable. We consider that well-designed, randomized, prospective studies comparing Allium RPS with other minimally invasive treatment methods are needed in the future.

## Conflicts of interest

There is no conflict of interest to declare for all authors.
